# Broadband and high-speed terahertz wireless sensing via vertical-transport Dirac-source detector

**DOI:** 10.1126/sciadv.aeg2196

**Published:** 2026-06-05

**Authors:** Xiaokai Pan, Yiming Wang, Hangxiang Wang, Zhen Hu, Yichong Zhang, Huichuan Fan, Yingdong Wei, Hongfei Wu, Zhaowen Bao, Xiaoyun Wang, Yan Huang, Xingjun Wang, Xiaoshuang Chen, Wei Lu, Lin Wang

**Affiliations:** ^1^ State Key Laboratory of Infrared Physics, Shanghai Institute of Technical Physics, Chinese Academy of Sciences, 500 Yu-tian Road, Shanghai 200083, China.; ^2^ University of Chinese Academy of Sciences, No. 19A Yuquan Road, Beijing 100049, China.; ^3^ Shanghai Frontiers Science Research Base of Intelligent Optoelectronics and Perception, College of Smart Materials and Future Energy and College of Future Information Technology and Institute of Optoelectronics, Fudan University, Shanghai 200433, China.; ^4^ School of Physics, Faculty of Basic Sciences, University of Shanghai for Science and Technology, Shanghai 200093, China.; ^5^ School of Physical Science and Technology, ShanghaiTech University, Shanghai 201210, China.

## Abstract

In the realm of wireless sensing, it is envisioned that sensing and communication functionalities will coexist and be fully integrated within a unified system. Future sensing systems thus necessitate detectors capable of operating at higher frequency bands—ranging from millimeter wave to terahertz (THz)—while delivering wider bandwidths, faster response rates, and enhanced functional integration. Dirac-source (DS) detectors use Dirac semimetals as hot-electron sources to capitalize on the low density of states (DOS) near the Dirac point, thereby effectively suppressing the formation of metal-induced gap states. Furthermore, when this architecture incorporates the inherent interlayer vertical electron transport of vertical van der Waals (vdW) heterostructures, it shows great promise for realizing low-power, post–Moore era sensing devices with superior injection and transport efficiencies. Here, we report a DS detector composed of the completely vertical gold/zirconium pentatelluride/graphene/gold structure (Au/ZrTe_5_/graphene/Au) heterojunction, which harnesses strong localized fields to achieve high thermionic emission. The detector manifests outstanding performance in terms of remarkable responsivity, exceeds a peak of 1600 V/W from 0.02 to 0.5 THz at room temperature, has a fast response time less than 20 ns, and notably is capable for heterodyne mixing with intermediate frequency (IF) bandwidth larger than ±26.5 gigahertz. Our results not only shed a fresh light on DS dynamics in the terahertz region but also highlight the transformative potential of semimetal electronics for applications in wireless energy harvesting, communication, and imaging.

## Teaser

A vertically stacked device detects terahertz waves with high sensitivity and nanosecond response.

## INTRODUCTION

The post–Moore era places higher demands on the sensor side in the field of wireless sensing. The advancement of modern semiconductor technologies has historically been largely driven by the device miniaturization, addressing the growing demand for faster, highly integrated systems (*1*–*4*). However, as the channel length decreases, conventional electronic devices such as transistors and diodes face intrinsic limitations including increased tunneling-induced channel resistance (*5*) and high parasitic capacitances (*6*), which hinder the maximum of operating frequency and further scaling. In recent years, the emergence of quantum topological two-dimensional (2D) semimetals has spurred extensive theoretical exploration in areas such as heterojunction dimensionality (*7*), band alignment, and metal/semimetal contacts (*8*–*11*). These quantum topological 2D materials showcase transport characteristics, attributed to their distinctive band structures and topological properties (*2*, *12*–*15*), leading to novel architectures and functionalities for semimetal-based electronics (*1*, *5*, *13*–*20*). Leveraging over ultralow contact resistance and high Fermi velocity, such materials hold promises for circumventing the limitations of conventional devices and advancing high-frequency electronic systems (*12*, *21*–*23*). This suggests that topological semimetals have the potential to meet the growing demands of wireless sensing technology for broader bandwidth, higher frequencies, faster speeds, and greater integration.

Among such, ZrTe_5_ has garnered considerable interests due to its large thermoelectric power (*24*), resistivity anomaly (*25*), and quantum oscillations (*26*, *27*) and manifests a few quantum revelations close to the phase boundary among a weak topological insulator, Dirac semimetal, and a strong topological insulator (*28*, *29*). Its metallic surface states and minimal bulk bandgap [on the order of tens of millielectron volts (meV); (*30*)] imply unconventional electrical behavior at metal/semimetal interfaces (*30*–*32*). The low density of states (DOS) in semimetals reduces Fermi-level pinning (FLP) and enables tunable Schottky barriers (SBs) with ultralow resistance (*2*). Moreover, linear energy dispersion facilitates ultrafast charge transport and high cutoff frequencies (*33*), while symmetry breaking induces transport phenomena such as the nonlinear Hall effect (*34*, *35*) and chiral magnetic effect (*36*, *37*). These properties can be harnessed to provide opportunities for energy-efficient Dirac-source (DS) detectors in high-frequency electronics (*2*, *38*, *39*).

**Fig. 1.**
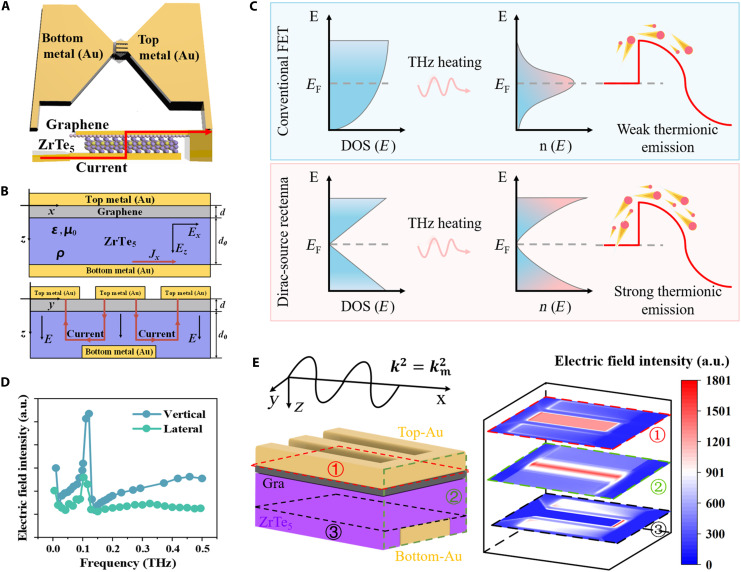
Architecture and field-enhancement mechanism of the ZrTe_5_/graphene DS detector. (**A**) Schematic of ZrTe_5_/graphene DS detector. The vertical-transport configuration of graphene and ZrTe_5_ is highlighted. (**B**) Side view of the ZrTe_5_/graphene DS detector: The upper diagram represents the *zx* plane, while the lower diagram represents the *zy* plane. (**C**) DOS versus electron energy for semiconductor (top) and semimetals (bottom). (**D**) Relationship between the electric field intensity and the incident THz frequency according to FDTD simulation, and green refers to lateral-transport heterojunction device while blue refers to vertical. (**E**) FDTD simulation at different cross-sections (the numbers represent the normalized electric field intensity). a.u., arbitrary unit.

Meanwhile, vertical stacked individual atomic layers provide a fascinating route for device integration (*40*), encompassing enhanced tunneling (*2*, *4*), ultrafast charge transfer (*41*), and quasiballistic electrical transport (*4*, *42*). In this study, the ZrTe_5_/graphene van der Waals (vdW) heterojunction for vertical-transport DS detector is designed with an embedded plasmonic cavity composed of top and bottom electrodes, as shown in Fig. 1A. This configuration enhances thermionic emission driven by broadband electromagnetic fields extending into the terahertz (THz) range, yielding a peak responsivity of 1600 V/W and an ultrafast response time below 20 ns, thereby surpassing state-of-the-art THz detectors in both performance and functionality. Furthermore, its tunability of nonlinear heterodyne mixing spans 20 to 340 GHz with a usable intermediate frequency (IF) bandwidth exceeding ±26.5 GHz, demonstrating its potential in millimeter wave/THz communication, imaging, and energy harvesting applications.

## RESULTS

### Deep subwavelength electromagnetic coupling of metallic cavity

Figure 1A provides a conceptual demonstration of the ZrTe_5_/graphene DS detector, highlighting the out-of-plane transport channel, which significantly reduces the channel length compared to lateral heterojunction structure and ease of fabrication. The shorter channels offer substantial advantages, including enhanced tunneling (*43*), ultrafast charge transfer–mediated device operation (*44*), and improved efficiency in thermal management and ohmic contact. The top electrode is decomposed into three fingers, and each finger is 6 μm in length and 2 μm in width, with a 1-μm gap between adjacent ones, as shown in Fig. 1B. This configuration facilitates transverse electromagnetic mode (TEM) propagating along the lateral direction in the deep-subwavelength regime (*45*).

As shown in fig. S1A, the resistance of our DS detector is ~1200 Ω and exhibits ohmic-like behavior. This property is in well accordance with the results shown in Fig. 1C that stronger thermionic current emerges due to the increased DOS of semimetal away from Dirac point, which allows for the efficient conversion of the received THz signals into electrical signals. To be more clear, the transport conductance is directly related to the energy-dependent electron density n(E) and can be expressed as $n\left(E\right)=\text{DOS}\left(E\right)\cdot f\left(E-E_{F}\right)$. Here, DOS is the DOS that grows up linearly with *E*, and $f\left(E-E_{F}\right)$ is the Fermi-Dirac distribution where $E_{F}$ is the Fermi level. Higher electron density means stronger thermionic emission, which results in stronger THz response (*46*). The thermionic behavior can be described as follows

$$J=A^{*}T_{e}^{\alpha }\text{exp}\left[-\frac{q\phi_{B}}{k_{B}T_{e}}\right]\left[1-\text{exp}\left(-\frac{\mathrm{qV}}{k_{B}T_{e}}\right)\right]$$ (1)

Here $A^{*}$ is the Richardson constant, $k_{B}$ is the Boltzmann constant, $T_{e}$ is electron temperature, α is an exponent equal to 1.5 for 2D materials, and *V* is the applied bias. It can be inferred from the relationship of Eq. 1 that stronger thermionic energy $k_{B}T_{e}$ produces higher photocurrent, and thus, strong optical field in submicrometer regime is indispensable.

For such a vertical-transport channel, the top and bottom electrodes of the detector form a metallic cavity that resembles a parallel-plate capacitor, where the alternating electric field from the THz waves received by the antenna induces a displacement current

$$I_{D}=\varepsilon \frac{\partial E}{\partial t}$$ (2)

where the displacement current *I*_D_ is caused by the time-varying electric field. The oscillating displacement current along the vertical direction generates the accumulated charges on two surfaces of electrodes that make up a metallic cavity, which confines the THz field within the subwavelength gap between metallic fingers and ultimately strengthens the rectification process. This mechanism explains the measurable rectified response under an alternating electric-field and the effect of metallic cavity.

The role of subwavelength propagation in rectification is further elucidated in the bottom panel of Fig. 1B. Figure 1E provides a magnified view of the metallic cavity, emphasizing the central part of the detector. The metal/graphene/ZrTe_5_/metal structure resembles a metal-semiconductor-metal configuration, which allows us to use a simplified model to capture the transport behavior of subwavelength TEM mode (*45*). This approach provides a straightforward method to optimize the device’s rectification performance as well as bandwidth.

Assuming the thickness of graphene layer is *d*, and the ZrTe_5_ layer is *d*_0_ with the conductivity ρ, and *d* << *d*_0_, using Maxwell’s equations for the ZrTe_5_ layer, and considering symmetry along the *y* axis, the following relationship is derived

$$\frac{\partial^{2}}{\partial x^{2}}E_{z}-\frac{\rho }{d_{0}}\frac{\partial }{\partial x}J_{x}+\xi^{2}E_{z}=0$$ (3)

Here, $E_{z}$ denotes the vertical component of the electric field at the barrier, while $J_{x}$ represents the current density within the semiconductor channel, and $\xi^{2}=\omega^{2}\mu_{0}\varepsilon$, with ω being the angular frequency. Given that ρ is nonzero and *d*_0_ is small on the order of tens of nanometers, the second term in Eq. *3* becomes dominant over the third term. Therefore, considering the continuity of current within the semiconductor layer, we arrive at

$$\frac{\partial^{2}}{\partial x^{2}}E_{z}=j\frac{\omega \varepsilon R_{\text{sh}}}{d_{0}}E_{z}$$ (4)

$R_{\text{sh}}=\rho /d_{0}$ is the sheet resistance of the ZrTe_5_ layer. Here, we obtain a dissipative mode characterized by a subwavelength oscillatory nature with a wavelength of $\lambda_{\text{sub}}$

$$\lambda_{\text{sub}}=2\pi \sqrt{\frac{2d_{0}}{\omega \varepsilon R_{\text{sh}}}}$$ (5)

Supposing the oscillation frequency is 0.3 THz, the required finger width along *y* axis can be retrieved. As depicted in the lower part of Fig. 1B, the discontinuity of top electrodes acting as oscillation ports sustains quasi-TEM mode oscillates with strong confinement of THz electric field. To ensure effective operation, the finger width must be smaller than the deep subwavelength limit of 2.75 μm as determined by Eq. 5, and the relationship between the deep-subwavelength limit and the maximum THz frequency is shown in fig. S1B. Each finger width of top electrodes in our design is 2 μm, which meets this criterion. To further understand the field profile, finite-difference time-domain (FDTD) simulations are carried out for the designed DS detector structure. The results are shown in Fig. 1 (D and E), and a maximum gain can be acquired at 0.1 THz. We conducted systematic simulation comparisons for lateral-transport heterojunction structures. The results demonstrate that, compared to lateral-transport structures, vertical-transport structures exhibit a significant enhancement in electric field intensity. More detailed information about the field distribution is provided in figs. S4 and S5. Also, cross-section view in Fig. 1E validates the strongest electric field occurring at the ZrTe_5_-graphene interface and allows for stronger THz photon absorption and thermionic emissions across the interface. In this context, the incorporation of metallic cavity in DS detector provides an efficient route to circumvent drawbacks in bandwidth and efficiency in traditional electronic devices.

### Broadband thermionic emission from millimeter wave to THz band

**Fig. 2.**
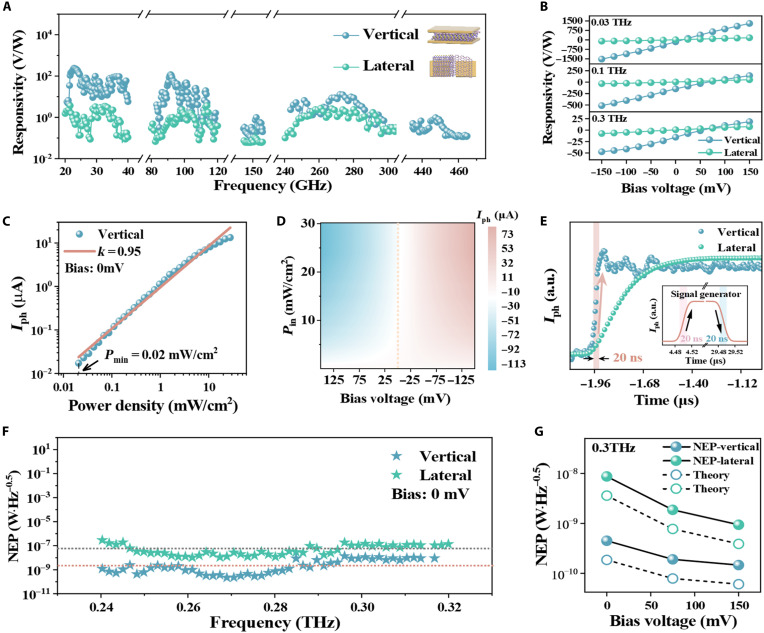
Performance benchmarking of vertical- and lateral-transport ZrTe_5_/graphene detectors. (**A**) The spectral dependence of the rectified photocurrent as obtained from lateral-transport heterojunction device (green) and vertical-transport heterojunction device (blue). The inset shows the experimental geometry. (**B**) Comparison of the bias dependence of responsivity at different frequencies (0.03, 0.1, and 0.3 THz) between vertical-transport heterojunction devices (blue) and lateral-transport heterojunction devices (green). (**C**) The power dependence of the photocurrent at zero bias–voltage condition. (**D**) Dependence of photocurrent on the incident optical power density at different bias voltages. (**E**) Comparison of the rise time between vertical-transport heterojunction devices (blue) and lateral-transport heterojunction devices (green), with the inset showing the intrinsic response time of the modulated signal. (**F**) Comparison of the NEP between vertical-transport heterojunction devices (blue) and lateral-transport heterojunction devices (green). (**G**) Comparison of the NEP between vertical-transport heterojunction devices (blue) and lateral-transport heterojunction devices (green) under different bias configurations, with the dashed lines representing theoretical results.

The performance of DS detector within peculiarly designed vertical-transport heterostructure is evaluated systematically under ambient conditions, with electromagnetic wave covering from 0.02 to 0.46 THz normally incident on the device and electric-field polarization parallel to the two sleeves of antenna. For testing purposes, the device was mounted on a printed circuit board, where the planar electrodes were spot-welded to the leads. As shown in Fig. 2A, the photocurrent exhibits a variation by an order of magnitude and is attributed to the substantial differences in incident power intensity and selective frequency response of metallic cavity effect. The detector achieves a responsivity of 250 V/W in the millimeter wave band (0.02 to 0.04 THz), 120 V/W in 0.08 ~ 0.12 THz, and 14 V/W in 0.24 to 0.28 THz range under zero bias, and the responsivity increases linearly with applied bias voltage as shown in Fig. 2B, which is unequivocally ascribed to the tunability of interface-barrier-height. Besides, the planar device with the same heterostructure and antenna sleeves but without metallic cavity exhibits much weaker responsivity and fails to detect signal beyond 400 GHz, validating the efficiency of proposed design.

Figure 2 (C and D) presents the power dependence under varied bias voltages, demonstrating a linear dynamic regime of 30 dB (the input power range in which the deviation of the output from its linear extrapolation does not exceed 1 dB is regarded as the linear operating range, whose upper limit is the 1-dB compression point), which begins to approach saturation at the 1-dB compression point, with the zero-crossing shifting toward positive voltage. To validate the response time of the detector, the electromagnetic wave source is transistor-transistor logic (TTL) modulated with an on/off time of ~20 ns, as shown in the inset of Fig. 2E, and the response is amplified using a high-speed current amplifier, DHPCA-100 (bandwidth: 200 MHz). By calculating the timescale of the switchable signal under fast on/off THz radiation modulation, it can be determined that the rise time of the detector is approaching the 20-ns limit of the TTL modulation, which is at least four orders of magnitude faster than that of other thermal-based detectors (*13*, *14*, *18*, *47*). The response time of lateral-transport heterojunction is about 10 times slower. Based on the above comparison, it can be concluded that vertical-transport heterojunctions underscore the superior performance for high-speed detection.

The variation of waveform versus power intensity, bias voltage, and THz radiation frequency (RF) is provided in fig. S1 (G to H). Once again, it is demonstrated that due to the antenna’s strong THz light coupling, the detector consistently maintains a stable waveform output across different frequency bands, bias voltages, and input power levels, indicating a large dynamic range.

Moreover, the sensitivity of the vertical-transport DS detector was evaluated using the noise equivalent power (NEP), which is defined as the ratio of noise density to responsivity, representing the minimum detectable power at a unity signal-to-noise ratio within a 1-Hz bandwidth (the measured noise spectral density is shown in fig. S1I). Figure 2F shows the NEP response at zero bias, and Fig. 2G further presents the bias-dependent NEP. The total noise current density can be obtained by the following formula

$$i_{n}=\frac{v_{n}}{r}=\frac{{\left(v_{t}+v_{b}\right)}^{1/2}}{r}={\left(\frac{4k_{B}T}{r}+2{\mathrm{qI}}_{d}\right)}^{1/2}$$ (6)

Here, $k_{B}$ stands for the Boltzmann constant, *T* is the temperature, *r* is the resistance of the detector, *q* is the elementary charge, and $I_{d}$ is dark current. At room temperature, the calculated noise current density ($i_{n}$) is approximately in the same order of magnitude as the results obtained from experimental measurements (Fig. 2G). The findings substantiate that the vertically integrated device has superior noise inhibition-ability, attributed to the asymmetric metallic contacting (*48*). Comparisons of noise power and NEP between conventional lateral- and vertical-transport heterojunction devices (Fig. 2F) highlight the advantages of the vertical-transport design. It is evident that, owing to the enhanced photo-response of the detector and the suppression of noise current under bias (see fig. S1I), an improvement of nearly one order of magnitude is achieved at a bias of 100 mV.

### Broadband heterodyne mixing from millimeter wave to THz band

**Fig. 3.**
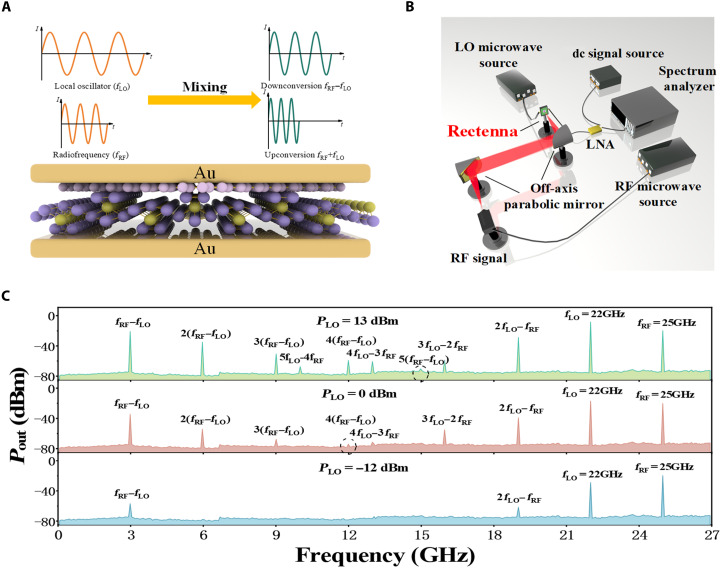
Heterodyne mixing based on the ZrTe_5_/graphene DS detector. (**A**) Schematic diagram of the mixing process. (**B**) Schematic illustration of the heterodyne mixing experiment setup. (**C**) Heterodyne mixing spectra (0 to 27 GHz) of LO (22 GHz) and RF (25 GHz) under different LO power levels (13, 0, and −12 dBm).

The fast response of ZrTe_5_/graphene vertical-transport DS detectors makes it particular appealing for heterodyne RF detection with an exceptionally high bandwidth. Frequency conversion and mixing are indispensable components in modern wireless communication systems (*49*–*51*), where the RF signals are converted into IF signals by mixing with a local oscillator (LO), while preserving the original information from the RF signals, as illustrated in Fig. 3A. The nonlinear thermionic emission characteristics of the detector govern the mixing process, which can be expressed as follows

$$I\left(V\right)=I\left(V_{0}\right)+\frac{\mathrm{dI}}{\mathrm{dV}}\left|{V=V_{0}}^{\left(V-V_{0}\right)+\frac{1}{2}\frac{d^{2}I}{{\mathrm{dV}}^{2}}}\right|{V=V_{0}}^{{\left(V-V_{0}\right)}^{2}+\cdots }$$ (7)

When the detector receives the LO signal and the RF signal, the voltage can be expressed as the superposition of these two signals, $V=V_{0}+V_{\text{RF}}\text{cos}\omega_{\text{RF}}t+V_{\text{LO}}\text{cos}\omega_{\text{LO}}t$. Here, $V_{\text{RF}}$ and $V_{\text{LO}}$ denote the amplitudes of the incident RF and LO signals, respectively, while $\omega_{\text{RF}}$ and $\omega_{\text{LO}}$ represent their corresponding angular frequencies. Substituting V into the above formula

$$\begin{array}{c} I\left(V\right)=I\left(V_{0}\right)+\frac{\mathrm{dI}}{\mathrm{dV}}|{V=V_{0}}^{\left(V_{\text{RF}}\text{cos}\omega_{\text{RF}}t+V_{\text{LO}}\text{cos}\omega_{\text{LO}}t\right)} \\ +\frac{1}{4}\frac{d^{2}I}{dV^{2}}|{V=V_{0}}^{\left[V_{\text{RF}}^{2}\left(\text{cos}2\omega_{\text{RF}}t-1\right)+V_{\text{LO}}^{2}\left(\text{cos}2\omega_{\text{LO}}t-1\right)\right]} \\ +\frac{1}{2}\frac{d^{2}I}{dV^{2}}|{V=V_{0}}^{\left[V_{\text{RF}}V_{\text{LO}}\text{cos}\left(\omega_{\text{RF}}+\omega_{\text{LO}}\right)t+V_{\text{RF}}V_{\text{LO}}\text{cos}\left(\omega_{\text{RF}}-\omega_{\text{LO}}\right)t\right]+\cdots } \end{array}$$ (8)

As a result, two new signal components are generated, with their respective frequencies being $\omega_{\text{RF}}+\omega_{\text{LO}}$ and $|\omega_{\text{RF}}-\omega_{\text{LO}}|$, associated with an upper sideband and a lower sideband signal. Figure 3A provides a conceptual view of the heterodyne mixing process, while Fig. 3B shows the experimental setup. Given the minimal ohmic loss in high resistive-Si, a backside illumination method is chosen for the LO signal.

The 2-mm wave/THz beams are focused onto the detector using off-axis parabolic mirrors and then converted into an electrical output at intermedia frequency and analyzed using a spectrometer (Agilent B2912A, 0 to 27 GHz). Figure 3C displays a typical heterodyne mixing spectrum, in which the LO is set at 22 GHz and the RF is set at 25 GHz. The frequency spectrum resulting from the mixing of the LO and RF signals reveals not only the presence of the IF but also the frequency-doubled components generated by the IF, such as $\pm (f_{\text{RF}}-f_{\text{LO}})$, $\pm 2(f_{\text{RF}}-f_{\text{LO}})$, $\pm 3(f_{\text{RF}}-f_{\text{LO}})$, $\pm 4\left(f_{\text{RF}}-f_{\text{LO}}\right)$, and $\pm 5(f_{\text{RF}}-f_{\text{LO}})$. In addition, the LO undergoes further mixing with both the IF and its higher harmonic generation, resulting in the generation of new frequency components, including $\pm (2f_{\text{LO}}-f_{\text{RF}})$, $\pm (3f_{\text{LO}}-2f_{\text{RF}})$, $\pm (4f_{\text{LO}}-3f_{\text{RF}})$, and $\pm (5f_{\text{LO}}-4f_{\text{RF}})$. Furthermore, by reducing the LO power, certain frequency components are observed to vanish, while the IF signal at 3 GHz maintains a high signal-to-noise ratio. This observation demonstrates that the detector holds a high dynamic range exceeding 45 dB in the microwave domain. A more detailed measurement of power dependence within the 0 to 27 GHz range is provided in fig. S6. The IF signal and other harmonic components exhibit relatively good linearity under LO or RF power variations up to 45 dB and begin to saturate at the 1-dB compression point.

**Fig. 4.**
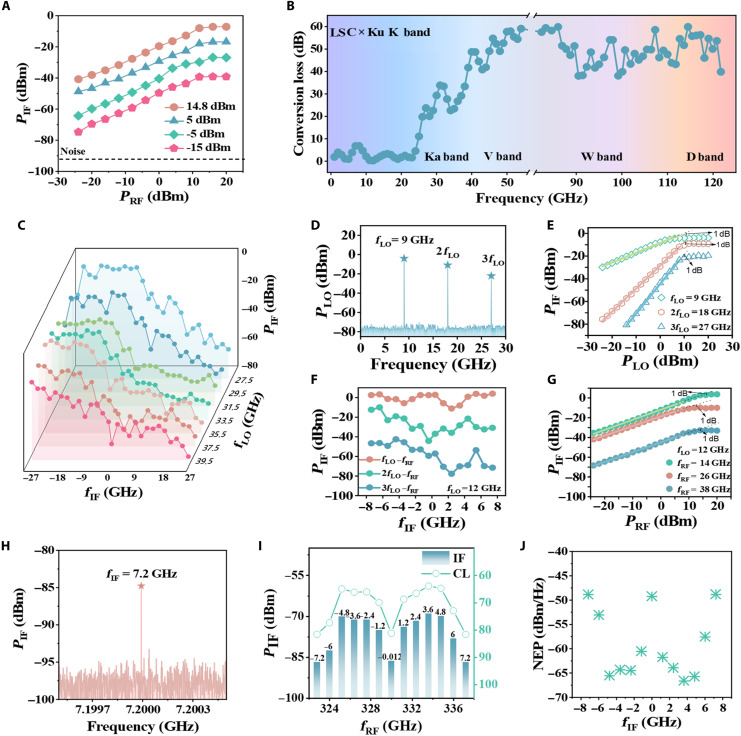
Wideband heterodyne conversion of the ZrTe_5_/graphene DS detector. (**A**) Relationship between IF power and RF power under different LO power configurations. (**B**) Frequency response of the conversion loss in 1 to 54 GHz and 82.5 to 120 GHz. (**C**) The up-conversion and down-conversion of the IF output signal across different RF frequencies covering the 1 to 66 GHz band, where −26.5 GHz < IF < 26.5 GHz with 26.5 GHz limited by the bandwidth of spectrum meter. (**D**) Harmonic spectrum of a single input signal frequency, including second and third harmonics. (**E**) Power-dependent relationships of the three different harmonic signals in (D). (**F**) Relationship between IF power and its frequency, with *f*_LO_ fixed at 12 GHz. The green curve represents the first harmonic heterodyne mixing, the red curve represents the second harmonic mixing, and the blue curve represents the third harmonic mixing. (**G**) With a fixed IF signal at 2 GHz, the variation of IF power as a function of RF power for *f*_RF_ − *f*_LO_, 2*f*_RF_ − *f*_LO_, and 3*f*_RF_ − *f*_LO_ mixing. (**H**) Spectrum for *f*_LO_ = 330 GHz and *f*_RF_ = 337.2 GHz, where *f*_IF_ = 7.2 GHz. (**I**) Histogram of the relationship between IF power/CL and RF frequency under the 330-GHz mixing configuration, where −7.5 GHz < IF < 7.5 GHz. The black axis represents IF output power, and the red axis represents CL. (**J**) The variation of NEP with *f*_IF_.

To further calibrate the dynamic range of the device, we monitored only the variations in the IF signal, while progressively reducing both the RF and LO power, as illustrated in Fig. 4A. When the LO power is reduced to −15 dBm and the RF power to −25 dBm, an IF signal can still be clearly resolved. The dynamic range of the detector encompasses both variations in incident power and changes in the RF frequency, effectively demonstrating the broadband capabilities of DS detector. Initially, within the microwave range of 0.5 to 66.5 GHz and with the LO frequency set to 40 GHz, the detector successfully covers an IF frequency range of ±26.5 GHz (limited by the spectrum analyzer’s bandwidth of 0 to 26.5 GHz, and potentially even higher). By calibrating the experimental setup, the IF signals also survive within the 80 to 120 GHz range. Notably, within higher frequency range, no further increase in IF signal loss was observed, as illustrated in Fig. 4B. The increased loss at frequency higher than 30 GHz is partly due to propagation losses of the outer-coupler and the broadening in the IF bandwidth, which reduces overall response. The formula for calculating mixing conversion loss (CL) is governed by

$$\text{CL}=10\times {\text{log}}_{10}\left(\frac{A_{\text{TIA}}\cdot P_{\text{input}}\cdot S_{\text{opt}}}{P_{\text{output}}\cdot S_{\text{source}}}\right)$$ (9)

where $A_{\text{TIA}}$ represents for the gain of an LNA (low-noise amplifier) (TIA). The above results indicate that the mixing frequency range of our detector is extendable into the THz regime and manifests superior performance with tunable bandwidth surpassing former developed ones.

To determine whether the detector can achieve a maximum IF bandwidth exceeding ±26.5 GHz under different LO frequencies, more detailed measurements were conducted, with the results displayed in Fig. 4C. Each spectrum depicts the variation of IF power for a specific LO frequency. It can be inferred that when RF frequency reaches 66 GHz (corresponding to LO of 39.5 GHz and IF of 26.5 GHz), the IF output at upper side band can maintain signal-to-noise ratio over 20 dB, despite the system loss increases as the frequency rises, indicating that the IF bandwidth can exceed ±26.5 GHz.

Given the excellent performance in heterodyne mixing, as well as the observed harmonic generation in Fig. 3C, we prefer to engineer the harmonic-mixing capabilities of our detector. Harmonic mixing holds substantial potential in future high-frequency communication, as it allows higher RF signals to be mixed with harmonics of the LO source, eliminating the burden on the requirement of high-precision, expensive signal source for LO. Thus, the high-frequency losses in the system’s route can be reasonably avoided, which greatly improves the detector’s capability at high-frequency domains. To validate the detector’s ability to generate harmonic signals, a 9-GHz signal was applied, and its second and third harmonics were observed on the spectrometer, as shown in Fig. 4D. Meanwhile, power conversion measurement was also performed, yielding results with strong linearity, as demonstrated in Fig. 4E. These findings confirm that the detector exhibits both odd and even number harmonic generation. To extend our analysis, the LO frequency was then adjusted to 12 GHz to evaluate broader harmonic mixing bandwidth. Harmonic mixing is conducted across the 16 to 32 GHz and 28 to 44 GHz bands, using the 24 and 36 GHz harmonic components generated by the LO as shown in Fig. 4F. Notably, these two frequency bands overlap partly, indicating that RF harmonic testing in the 28 to 32 GHz range is influenced by both second- and third-order harmonic mixing. Last, a variable power calibration was conducted on harmonic mixing, and the results are given in Fig. 4G, which demonstrates excellent 40-dB linearity and the harmonic mixing capability of the detector. Our finding suggests that mixing can be extended from millimeter wave to the THz range, paving the way for future high-bandwidth communication/imaging applications.

Moreover, to fully explore the capability of the detector to harvest higher-frequency signals, we further used two 340-GHz THz sources for higher frequency mixing. The IF signal obtained on the spectrum analyzer is plotted in Fig. 4H, where the LO signal is set as 330 GHz, while the RF frequency ranges from 322.8 to 337.2 GHz. The calculated CL and NEP are presented in Fig. 4 (I and J), respectively, where the NEP can be obtained by the following formula (*52*)

$$\text{NEP}=N_{F}+P_{\text{input}}-P_{\text{output}}$$ (10)

Here, $N_{F}$ is noise floor about −95 dBm/Hz, and a CL of 65 dB and an NEP of −65 dBm/Hz can be derived. Although these values remain inferior to the CL and NEP of Schottky diode mixers, the DS detector demonstrates higher bandwidth and superior performance than graphene field-effect transistor (G-FET). Given that the device’s strong thermionic emission is largely frequency-independent, it is suggested that the DS detector holds substantial potential for higher-frequency applications.

### Heterodyne wireless communication and high-sensitivity heterodyne imaging

**Fig. 5.**
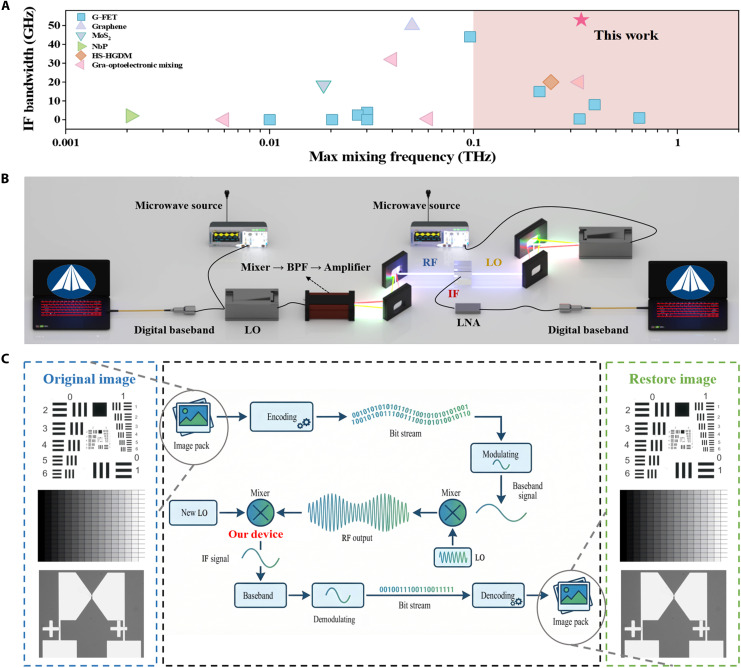
W-band THz heterodyne communication enabled by the vertical transport DS detector. (**A**) IF bandwidth comparison of different heterodyne mixers. HS-HGDM refers to half-subdivision and half-global design method. (**B**) Schematic diagram of a W band–focused THz heterodyne communication link based on a vertical transport DS detector. (**C**) Heterodyne communication encoding and decoding scheme. Three categories of representative test images were selected as transmission targets, covering three evaluation dimensions: “resolution characterization,” “grayscale reproduction capability,” and “practical scene application.” The blue dashed box on the left indicates the original images, while the blue dashed box on the right shows the images recovered after transmission.

Furthermore, Fig. 5A summarizes the mixing performance of the ZrTe_5_-graphene detector (*16*, *21*, *53*–*68*), showing up superiorities in mixing frequency and IF bandwidth compared with other devices derived from 2D materials such as graphene, MoS_2_, etc.

THz technology is among the most important enabling platforms for next-generation wireless communications. Compared with direct detection, heterodyne detection provides markedly higher receiver sensitivity. Leveraging the mixing-enabled detection capability of our vertical-transport DS detector, we constructed a heterodyne communication link (Fig. 5B) to evaluate device-enabled wireless image transmission.

Both the transmitter and receiver used a digital RF module based on an STC8A8K64D4 microcontroller. The module supports bidirectional transparent transmission, automatic packetization, and automatic retransmission upon packet loss, and it can generate a 2.4-GHz baseband RF signal (used here as the IF). At the transmitter, an open-source visual communication software package was first used to encode the image packet (image pack) into a digital bit stream. The data rate was set to 2 Mbps. On-off keying (OOK) was used to modulate the bit stream onto a 2.4-GHz carrier, where the presence (“on”) and absence (“off”) of the carrier represent binary “1” and “0,” respectively.

The modulated IF was then up-converted using a W-band mixer. The mixer combined the modulated IF with a 97-GHz LO via a waveguide, generating RF sidebands at 94.6 and 99.4 GHz. A band-pass filter (85 to 95 GHz) selected the 94.6-GHz component, which was subsequently amplified by a W-band power amplifier (PA) with a gain of 20 dB and radiated through a horn antenna.

At the receiver, the vertical-transport DS detector served as the mixer, down-converting the 94.6-GHz RF signal with the 97-GHz LO to a 2.4-GHz IF. The IF was amplified by an LNA and fed into a digital baseband modem. The modem demodulated the amplitude modulation/OOK signal via envelope detection (i.e., by tracking the envelope corresponding to amplitude variations) to recover the original digital data. As shown in Fig. 5C, we selected three categories of images with typical testing significance as transmission targets, covering the three testing dimensions of “resolution characterization,” “grayscale reproduction capability,” and “practical scene application.” All are standard test images in the field of THz communications. Last, the transmitted content was reconstructed using the open-source visual communication software, completing information reception and image recovery.

To quantitatively characterize the image transmission quality of the THz heterodyne communication link, we used peak signal-to-noise ratio (PSNR), pixel matching rate, and bit error rate (BER) to evaluate the system’s transmission performance. Experimental results show that the PSNR for standard test images transmitted over this communication system consistently exceeds 15 dB, meeting the signal-to-noise ratio threshold (≥10 dB) required for normal operation of digital communication systems. The pixelwise matching rate is above 99.5%, with only negligible minor pixel deviations observed in localized noisy regions. At a transmission rate of 2 Mbps, the system’s BER remains stable below 10^−6^, providing reliable support for end-to-end data transmission. These quantitative results fully demonstrate that the THz communication link based on a vertical-transport DS detector has excellent image reconstruction capability and data transmission reliability.

To further verify the operational stability and environmental adaptability of the communication system, we evaluated system performance under varying data rates and transmission distances (see fig. S9). In data rate testing, we selected four representative rates—1, 2, 2.5, and 3 Mbps—for comparative experiments. The results indicate that the system maintains stable operation in terms of signal waveform, PSNR, and BER at rates up to 2 Mbps. In transmission distance testing, we examined four short-range scenarios: 0.5, 1.0, 1.5, and 2 m. The results show no significant signal distortion within a distance of 1.5 m, fully validating the stability and practicality of the system for short-range THz wireless communication applications.

Moreover, we slightly modified the above communication link to build a THz heterodyne imaging system, in which the target sample was imaged via step-and-scan acquisition (fig.S7A). The LO signal (330 GHz) and the RF signal (334 GHz) were collimated and simultaneously incident on the vertical-transport DS detector. Compared with THz direct imaging, heterodyne imaging benefits from the high responsivity, markedly improved signal-to-noise ratio, and ultrafast response of the vertical-transport DS detector, enabling a larger dynamic range and a lower detectable power. As a result, the system sensitivity is improved by more than 10 dB (fig. S7B). This substantial enhancement highlights the superior sensitivity and resolution of heterodyne imaging, making it particularly attractive for detecting subtle variations in the sample.

## DISCUSSION

In conclusion, we have developed an innovative DS detector based on a metal/semimetal vertical-transport heterojunction that host versatile functions as both a detector and a mixer. This detector achieves remarkable performances, including an ultrafast response time of 20 ns, high sensitivity of 1600 V/W, and a broadband response of 0.465 THz from microwave to the THz range. These results demonstrate the advantages of metal/semimetal contacts, such as reduced FLP, minimal SB effects at graphene interfaces, and robust thermionic emission, showcasing the superior interface properties and functionality of this design. In terms of heterodyne mixing, the detector operates effectively over ±26.5-GHz IF bandwidth with dynamic range exceeding 40 dB, as followed by both odd and even harmonic mixings, demonstrating substantial potential for advanced mixer operation. Leveraging on its broadband-mixing capability spanning from the microwave to the THz regime, we successfully demonstrated W-band heterodyne wireless communication as well as heterodyne imaging at 340 GHz. This approach reduces the performance burden on THz detectors compared to conventional routes, offering a pathway for the development of scalable THz technologies. This study establishes DS detectors as a promising platform for rectifiers and mixers based on advanced quantum topological materials, with potential applications in communication, imaging, and energy harvesting systems.

## MATERIALS AND METHODS

### Device architecture

Our vertical-transport DS detector is fabricated through a sequential vdW assembly process. Single-crystalline ZrTe_5_ flakes and monolayer graphene (chemical vapor deposition-grown) are mechanically exfoliated and deterministically transferred onto a prepatterned-bottom Au electrode on a high-resistivity silicon substrate. A top Au electrode featuring a three-finger antenna structure is then deposited onto the graphene layer, forming the complete metal/graphene/ZrTe_5_/metal stack. The electrodes were patterned by electron-beam lithography.

### Rectification characterizations

The electrical characteristics of the devices were measured at room temperature (300 K) using a digital source meter analyzer (Agilent B2912A). A W-band PA in conjunction with a Virginia Diodes Inc. (VDI) Tripler (Waveguide Rectangular 2.8) was used to generate radiation within the frequency range of 0.25 to 0.30 THz. In addition, a microwave source (Agilent E8257D, 0.02 to 0.04 THz) connected to a VDI multiplier (WR 9.0) was used to produce radiation within the frequency range of 0.07 to 0.12 THz. A 435 to 465 GHz transmitter module with a 36-fold frequency doubling scheme was used. In this configuration, the initial microwave signal undergoes multiple stages of frequency-doubling amplification to generate a D-band millimeter-wave signal. This millimeter-wave signal serves as an LO, driving the 435 to 465 GHz triplexer. To ensure accurate measurements, the output power of the THz radiation was carefully calibrated using a TK100 power meter. The dc voltage, denoted as $V_{\text{dc}}=2.2G_{n}\Delta uR$ is directed through the current preamplifier and subsequently measured by the lock-in amplifier, where the factor is the Fourier component of the square wave modulation, $G_{n}$ is the gain factor of the current preamplifier, Δu is the voltage signal from the lock-in amplifier, and ***R*** is the resistance of the device. The overall system was aligned by the polarizer, and the angle of rotation is adjusted precisely with a high-precision angle holder.

### Response time characterizations

For high-frequency modulated signals, the built-in modulation bandwidth of the microwave source (E8257D) is insufficient. Therefore, we use an ultrafast modulator to modulate the THz signal generated by the Frequency Tripler (VDI). At the output of the device, we replace the current preamplifier with a higher-bandwidth (200 MHz) model (DHPCA-100) for signal amplification. The modulated signal is then captured using a lock-in amplifier (SR830) and subsequently read via an oscilloscope to obtain the waveform.

### Heterodyne-mixing characterizations

In mixing test, we present the experimental process concept as illustrated in Fig. 3B. Due to different THz frequency bands, different THz source configurations are required for direct/heterodyne detection. For microwave mixing in the 0 to 66.5 GHz range, as shown in Fig. 4C, two microwave sources (Agilent E8257D, 0.02 to 0.04 THz) were used to provide the LO and RF signals, respectively. For the frequency-mixing configuration in the 0.08 to 0.12 THz band, as shown in Fig. 4B, an Agilent E8257D microwave source (0.02 to 0.04 THz) was connected to a VDI multiplier (WR 9.0) along with a 100-GHz source. For frequency mixing in the 0.32 to 0.34 THz range, two W-band PAs were used in conjunction with a VDI Tripler (WR 2.8). Regarding the high-order harmonic mixing portion, we use the LO signal to generate high-order harmonic signals at the incident point. Subsequently, these generated signals are mixed with another signal, RF, producing an IF signal denoted as (*nf*_LO_ − *f*_RF_). This resultant signal is then amplified through the LNA and detected by the spectrum analyzer.

## Supplementary Materials

### This PDF file includes:

Notes S1 to S6 Figs. S1 to S9
