# Supplementary material for: Broadband and high-speed terahertz wireless sensing via vertical-transport Dirac-source detector
Source: Sci Adv. 2026 Jun 5;12(23):eaeg2196. doi: 10.1126/sciadv.aeg2196 (PMC13240216; doi:10.1126/sciadv.aeg2196)
Supplement: Supplementary file 1 — Notes S1 to S6 Figs. S1 to S9 [file sciadv.aeg2196_sm.pdf]

Science Advances
AAAS

Supplementary Materials for

Broadband and high-speed terahertz wireless sensing via vertical-transport Dirac-source detector

Xiaokai Pan *et al.*

Corresponding author: Lin Wang, wanglin@mail.sitp.ac.cn

Sci. Adv. **12**, eaeg2196 (2026)
DOI: 10.1126/sciadv.aeg2196

This PDF file includes:

Notes S1 to S6
Figs. S1 to S9

Supplementary note 1: THz characterization

The current-voltage (I/V) characteristics of the terahertz detector are shown in Supplementary Fig. S1a. In the figure, the orange curve represents the I/V characteristics under ohmic contact conditions, while the green curve depicts the I-V response measured under 1 kHz modulated microwave irradiation, demonstrating photo-response features. The actual structure of the detector is illustrated in the inset at the bottom right. The detector exhibits an excellent response to microwave bias, achieving a responsivity of 1524 V/W, which allows the microwave-modulated I-V curve to be clearly observed in the figure. In Supplementary Fig. S1b, we estimated the relationship between the dimensions of the interdigitated channel and the subwavelength characteristics based on Equation 5. Here, d0 represents the thickness of $ZrTe_5$, measured using AFM. The dielectric constant is approximately 10 times that of the vacuum permittivity. Near room temperature, the resistivity of $ZrTe_5$ may increase compared to its low-temperature value, typically around 0.1 Ω·cm, which enables the derivation of the relationship between wavelength and detection frequency, as shown in Supplementary Fig. S1b.

Simulation Details: ① Antenna dimensions — The top Au electrode features a three-finger structure with a finger length of 6 μm, finger width of 2 μm, and finger spacing of 1 μm. The substrate consists of a high-resistivity silicon/silicon dioxide stack. The antenna metal is Cr/Au. For the horizontal antenna, a 10 nm Cr / 30 nm Au layer is used; for the vertical antenna, the bottom electrode is 10 nm Cr / 20 nm Au, and the top electrode is 10 nm Cr / 30 nm Au. ② Excitation source parameters— Plane wave excitation is employed with polarization parallel to the antenna sleeve, and the incident frequency ranges from 0.02 to 0.5 THz. ③ Simulation software and boundary conditions — Simulations are performed using FDTD Solutions software, with boundary conditions set to perfectly matched layer (PML) to avoid electromagnetic wave reflection interference. ④ Mesh resolution — A sub-micrometer mesh grid with a size of 50 nm is adopted to ensure simulation accuracy.

The polarization characteristics of the antenna were also simulated using FDTD Solutions during the simulation of the vertical junction channel. As shown in Supplementary Fig. S1c, the overall polarization pattern exhibits an "8"-shaped structure. The polarization characteristics at different frequencies are presented in Supplementary Fig. S3. After the device fabrication was completed, we experimentally evaluated the overall polarization of the device by introducing a terahertz polarizer between the horn antenna and the device. The corresponding results are shown in Supplementary Fig. S1d.

In terahertz rectification experiment, we modulated the 0.32 THz incident signal with a square wave (on/off) at various modulation frequencies (up to 1 MHz). This causes the terahertz wave to switch between high and low intensity at the modulation frequency, and the rectified photocurrent carries that modulation frequency (detected via a lock-in amplifier). However, as the modulation frequency increases, the device cannot fully follow the rapid on/off switching due to its finite response speed. As a result, the amplitude of the measured photocurrent gradually decreases at higher modulation rates, as shown in Supplementary Fig. S1e, the modulation frequency corresponding to the 3 dB point, where the photocurrent intensity decreases by half, is 1.3 MHz. This indirectly demonstrates the device's stability in receiving terahertz waves at different modulation frequencies, as well as its ultrafast response time.

The dependence of the waveform on power intensity, bias voltage, and terahertz radiation frequency is presented in Supplementary Figs. S1f, S1g, and S1h. These results further confirm that, owing to the strong terahertz coupling of the antenna, the rectenna consistently maintains a stable waveform output across various frequency bands, bias voltages, and input power levels, demonstrating a broad dynamic range. Supplementary Fig. S1i illustrates the relationship between noise current and modulation frequency under different bias configurations. As the bias voltage increases, the noise current also rises; however, the responsivity improves by several orders of magnitude. Consequently, the noise equivalent power (NEP) decreases at higher bias voltages.

Supplementary note 2: The measurement method of heterodyne mixing

In the measurements, off-axis parabolic mirrors (OAPMs) were employed to focus two incident beams onto the device surface, and their polarization directions were aligned with the on-chip antenna to maximize the coupling efficiency. The intermediate frequency (IF) was chosen within the bandwidth of the readout link for measurements.

Signal Source and Mixing Link

Microwave band (2–60 GHz): The local oscillator (LO) signal was provided by an Agilent E8257D microwave source (250 kHz–40 GHz), and the radio frequency (RF) signal was output by a Ceyear 3672E vector network analyzer (VNA, 10 MHz–67 GHz).

Sub-terahertz (sub-THz) band (80–120 GHz): The LO signal was supplied by two THz IMPATT sources operating at 96 GHz and 100 GHz, respectively. The RF signal was generated by a microwave source followed by a WR3 tripler (80–120 GHz).

Terahertz (THz) band (320–330 GHz): The RF and LO signals were provided by a 12× frequency multiplier chain (320–340 GHz) and a 24× frequency multiplier chain (330–355 GHz), respectively.

Readout and Data Acquisition

The device output was first amplified by a low-noise amplifier (LNA, bandwidth: ~50 kHz–20 GHz) and then fed into an Agilent E4407B spectrum analyzer. When acquiring mixed-frequency spectral lines, the resolution bandwidth/video bandwidth (RBW/VBW) and averaging times were properly configured to balance spectral resolution and noise floor, while avoiding overload of the LNA or spectrum analyzer. Band-pass or low-pass filters were adopted when necessary to restrict the IF bandwidth. If the noise floor was elevated, a bias-tee was used for DC/RF separation.

The test procedure was as follows: The RF frequency was swept at a fixed LO power, and the variation of the IF amplitude with IF frequency points was recorded to obtain the IF bandwidth. Subsequently, the RF and LO powers were varied, and the linear operating region and linear dynamic range were evaluated under a predefined distortion threshold.

Supplementary note 3: Heterodyne imaging system

System and Quasi-Optical Configuration: As shown in Fig. S7, we constructed a terahertz imaging system capable of operating in both direct detection and heterodyne detection modes. The orange optical path corresponds to the direct detection configuration: the terahertz beam is focused by a pair of off-axis parabolic mirrors onto the sample mounted on a two-dimensional stepping translation stage, and the transmitted beam is collected by another pair of parabolic mirrors and focused onto the detector. By raster scanning the imaging target in a plane perpendicular to the beam propagation direction, a point-by-point scanning image of 100 × 100 pixels is obtained, with a step size matching the spot diameter. During the experiment, it is essential to ensure alignment between the polarization direction of the terahertz beam and the antenna polarization, and to adjust the beam focus to coincide with the sample plane to maximize coupling efficiency.

Principle and Implementation of Heterodyne Imaging: The system operates in heterodyne mode after incorporating an additional pair of parabolic mirrors to couple the local oscillator (LO) terahertz beam. The radio frequency (RF) beam is amplitude-modulated at a preset low modulation frequency, and a lock-in amplifier performs phase-sensitive detection using this modulation as a reference. The LO beam is left unmodulated and therefore is not directly detected by the lock-in amplifier. The intermediate frequency signal generated through mixing inherits the modulation applied to the RF, enabling high-signal-to-noise-ratio readout by the lock-in amplifier. Compared with direct detection, which measures only the RF signal, heterodyne detection significantly enhances the effective signal strength and detection sensitivity through controlled LO amplification and frequency downconversion, resulting in higher imaging contrast and improved image quality.

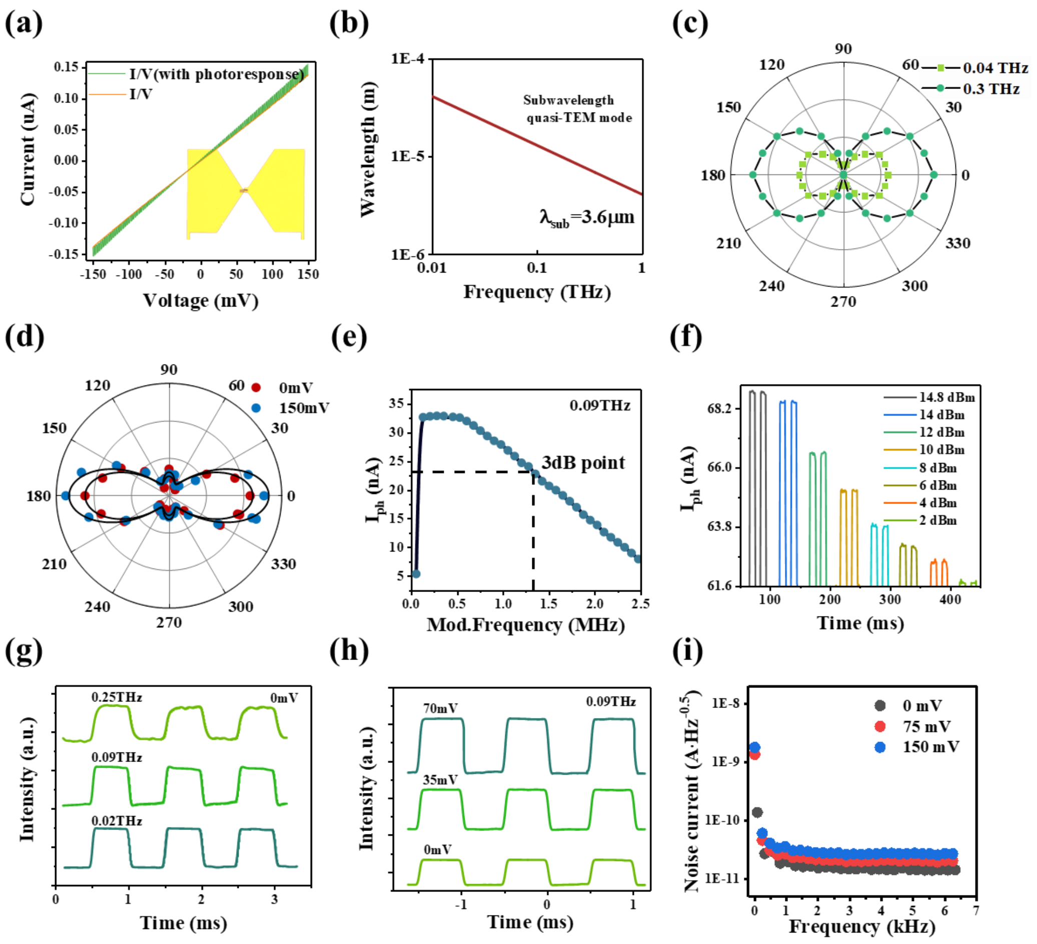

Supplementary Figure S1. (a) The Ohmic-like characteristic of the rectenna (orange) and the IV curve of the photocurrent response modulated by external microwave irradiation (green). (b) Wavelength versus frequency of subwavelength quasi-TEM mode. (c) Antenna Polarization Characteristics from FDTD Simulation at 0.04 THz and 0.3 THz. (d) Bias-Dependent Photocurrent Angle-Resolved Spectrum at 0 mV and 150 mV Bias Voltages. (e) Photocurrent variation with modulation frequency, with a 3 dB point at 1.3 MHz. (f) Waveform under different Incident THz power. (g) Waveform under different incident THz frequencies at 0 mV bias voltage. (h) Waveform variation as a function of bias voltage, with a fixed incident terahertz frequency of 0.09 THz. (i) The comparison of noise currents between different voltage bias.

Supplementary note 4: material characterization

To ensure the high crystalline quality of $ZrTe_5$, the material was characterized prior to device fabrication. The atomic structure of $ZrTe_5$ is depicted in Fig. S2a, with the inset providing a side view, revealing its layered structure along the y-axis. Fig. S2b presents the FFT diffraction pattern captured from the bulk region. To further evaluate the material's quality, spherical-aberration corrected scanning transmission electron microscopy (STEM) was employed to analyze the composition of $ZrTe_5$, as illustrated in Fig. S2c. The EDX elemental mapping images (Supplementary Fig. S2d and S2e) demonstrate a uniform distribution of elements within $ZrTe_5$. Additionally, atomic force microscopy (AFM) images (Supplementary Fig. S2f) and Raman spectra (Supplementary Fig. S2g) indicate the high purity of the sample, with the positions and vibrational modes of the six Raman peaks aligning well with previous reports.

The vertical $ZrTe_5$/graphene heterojunction is designed based on a self-mixing mechanism. The vertical structure is fabricated by performing photolithography twice on a high-resistance silicon wafer to create the electrodes. After the bottom electrode is patterned, $ZrTe_5$ and graphene are mechanically exfoliated and sequentially transferred onto the bottom electrode channel using a dry transfer technique. Special care is taken to ensure that the graphene covers as much of the $ZrTe_5$ as possible without contacting the bottom electrode. Next, a top electrode is patterned over the graphene through photolithography, ensuring the alignment of the top and bottom electrode channels is as perpendicular as possible to the surface of the high-resistance silicon wafer, thereby forming the vertical heterojunction. The bottom electrode consists of 10 nm Cr and 10 nm Au, while the top electrode consists of 10 nm Cr and 40 nm Au. Once the antenna structure is completed, the drain and source of the detector are wire-bonded to the coplanar waveguide (CPW) and ground on the printed circuit board (PCB), respectively.

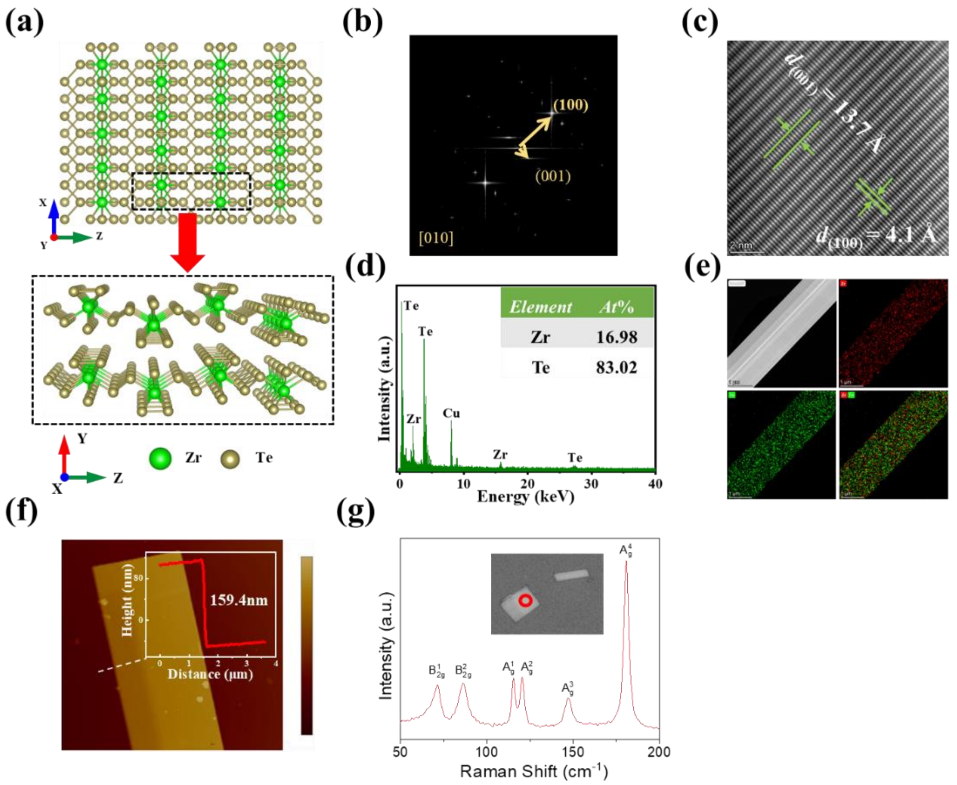

Supplementary Figure S2. (a) Atomic structure of the $ZrTe_5$ flake. The gray and green spheres denote Te and Zr atoms, respectively. (b) Selected-area electron diffraction patterns captured from the bulk area. (c) Zoomed-in atomic-resolution spherical-aberration-corrected STEM images of the bulk part from (b). (d) XRD analysis of the $ZrTe_5$ material confirms its crystalline structure, with the atomic ratio of zirconium (Zr) to tellurium (Te) measured to be 1:5. (e) Low-magnification TEM image of $ZrTe_5$ sample and corresponding EDS elemental mapping. (f) Corresponding AFM image of $ZrTe_5$ crystal transferred on SiO2/Si substrate. (g) Raman spectroscopy on $ZrTe_5$ sample.

Supplementary note 5: Electric field patterns in $ZrTe_5$-graghene vertical heterojunction rectenna.

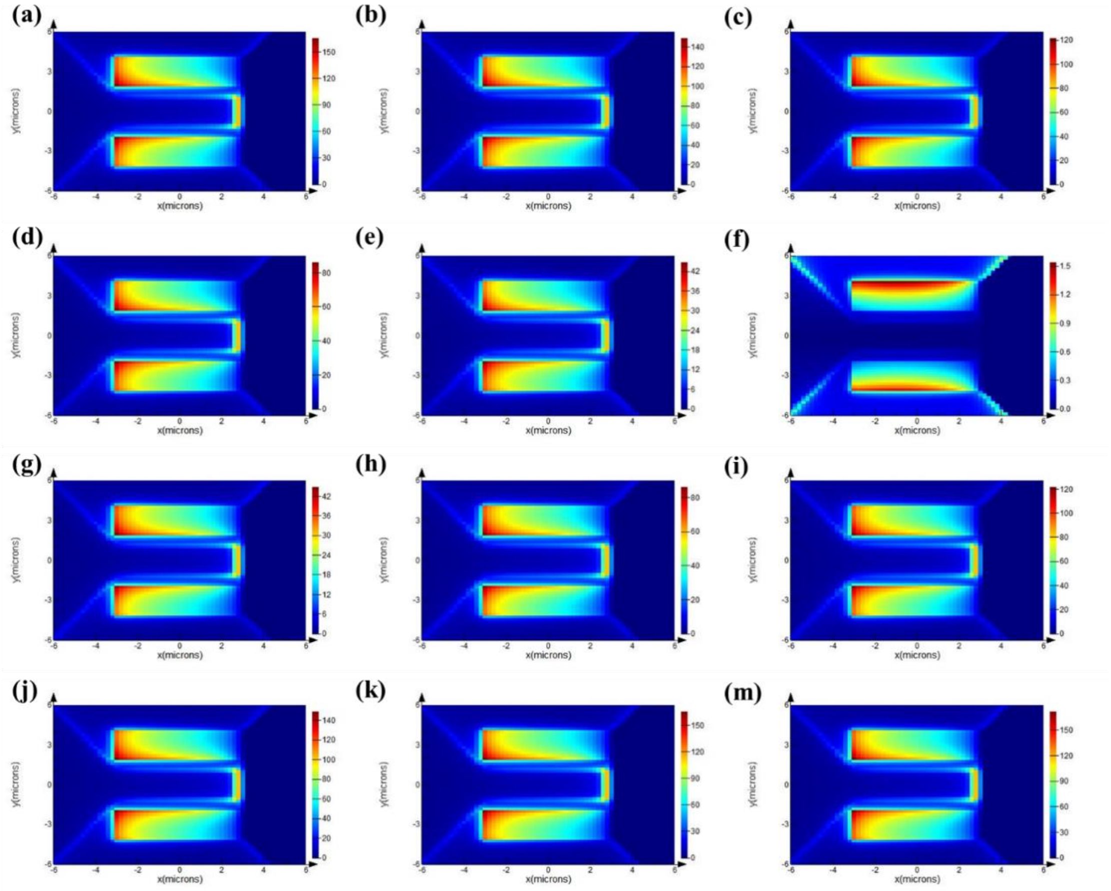

Supplementary Figure S3. Simulation of an electronic rectenna operating at a terahertz frequency of 0.1 THz under different polarization conditions. All colored values represent the normalized electric field intensity. (a) Polarization at 15°, (b) 30°, (c) 45°, (d) 60°, (e) 75°, (f) 90°, (g) 105°, (h) 120°, (i) 135°, (j) 150°, (k) 165°, and (m) 180°.

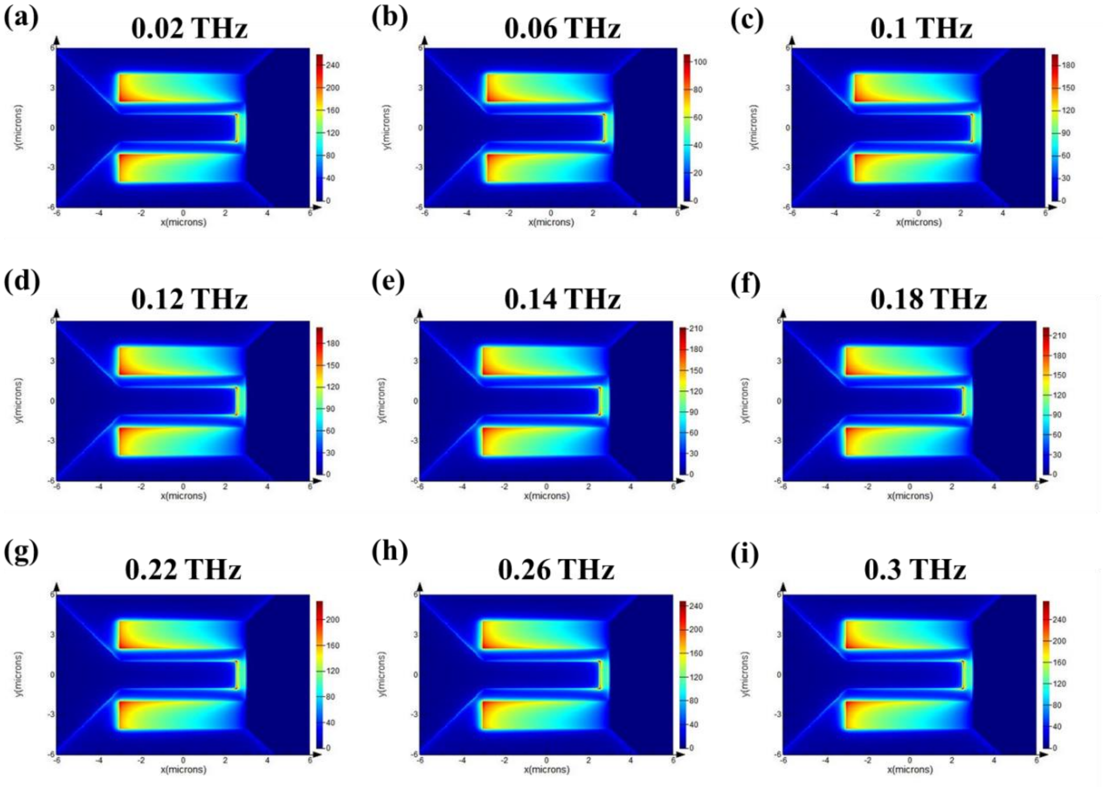

Supplementary Figure S4. Simulation of an electronic rectenna ($ZrTe_5$-graphene vertical heterojunction) operating at different frequencies (0.02-0.3 THz). All colored values represent the normalized electric field intensity. (a) Frequency of 0.02 THz, (b) 0.06 THz, (c) 0.1 THz, (d) 0.12 THz, (e) 0.14 THz, (f) 0.18 THz, (g) 0.22 THz, (h) 0.26 THz, and (i) 0.3 THz.

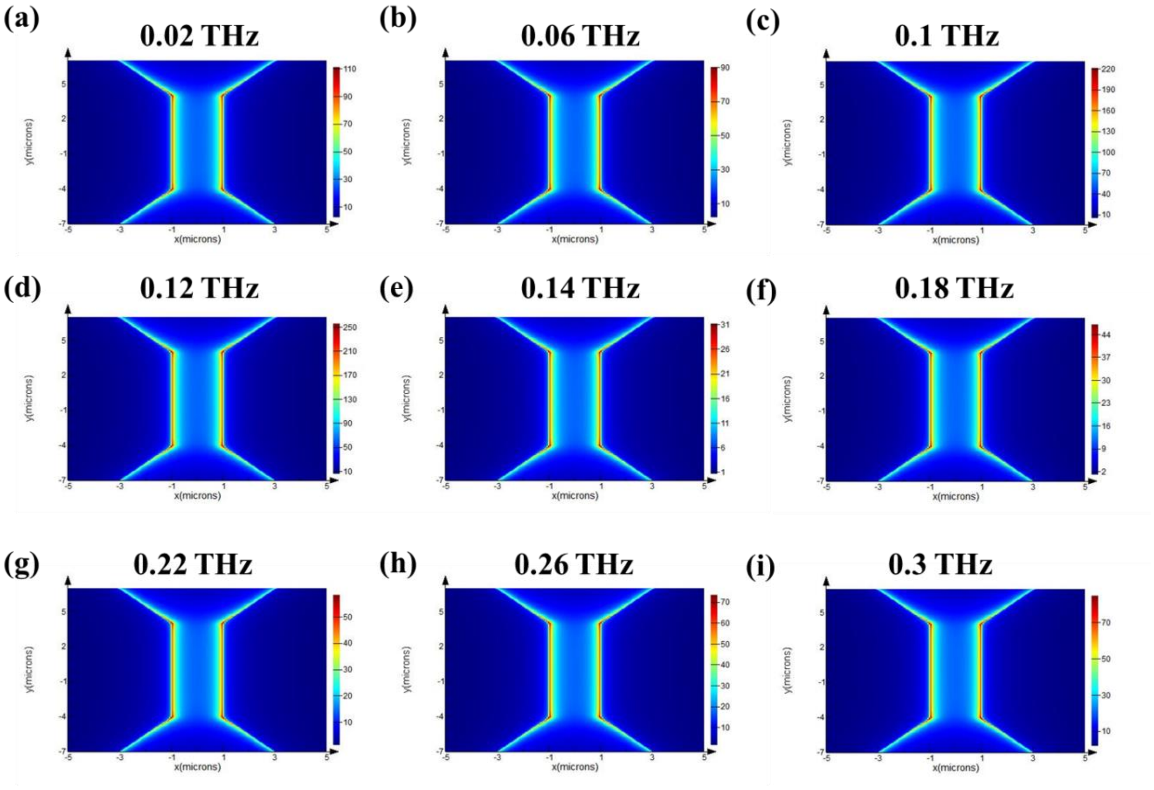

Supplementary Figure S5. Simulation of an electronic rectenna ($ZrTe_5$-graphene non-vertical heterojunction) operating at different frequencies (0.02-0.3 THz). All colored values represent the normalized electric field intensity. (a) Frequency of 0.02 THz, (b) 0.06 THz, (c) 0.1 THz, (d) 0.12 THz, (e) 0.14 THz, (f) 0.18 THz, (g) 0.22 THz, (h) 0.26 THz, and (i) 0.3 THz.

Supplementary note 6: Heterodyne mixing in microwave

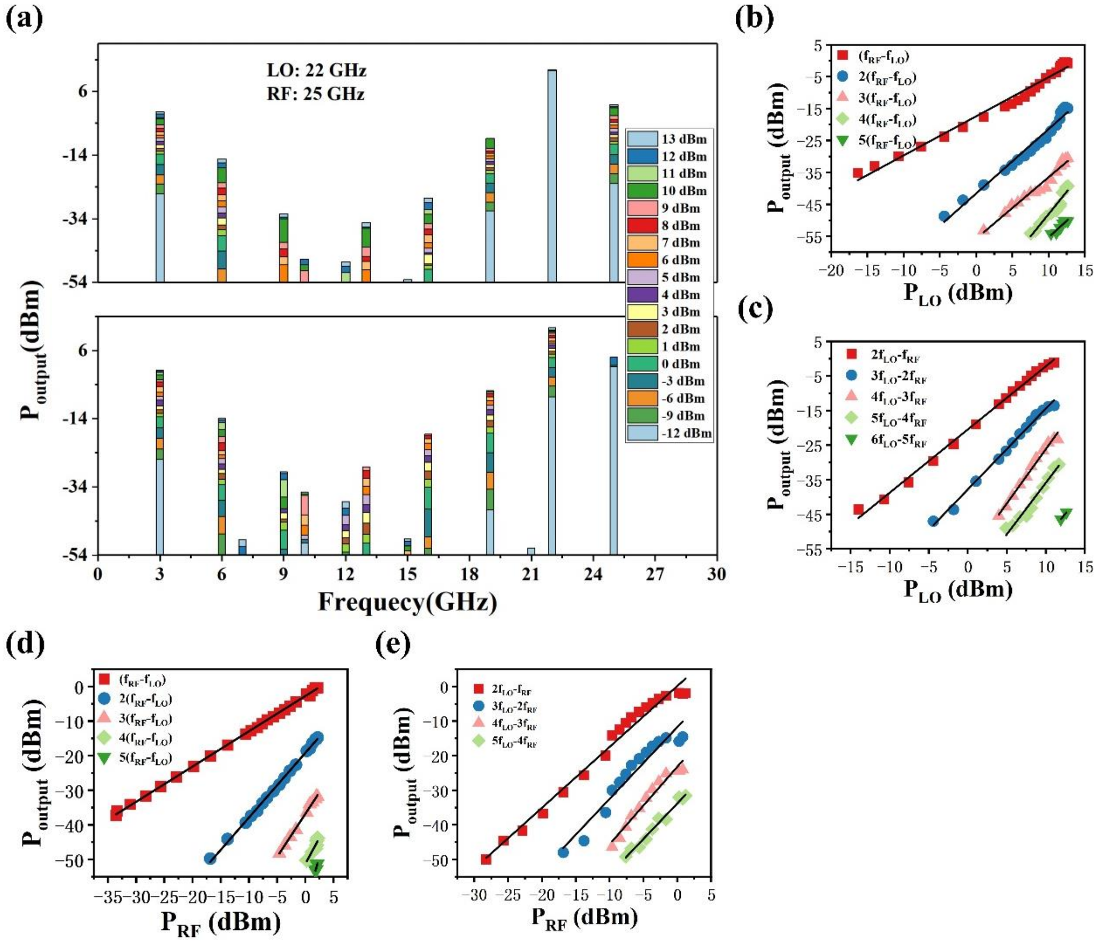

Supplementary Figure S6. (a) The upper panel illustrates the power variations of intermediate frequency (IF) and harmonic signals under different RF power levels for the mixing of LO (22 GHz) and RF (25 GHz) signals within the 0-27 GHz frequency range on a spectrum analyzer. The lower panel presents the power variations of IF and harmonic signals as a function of LO frequency and power. (b) The dependence of IF and its harmonics on LO power. (c) The variation of second-order mixing harmonics with LO power. (d) The dependence of IF and its harmonics on RF power. (e) The variation of second-order mixing harmonics with RF power.

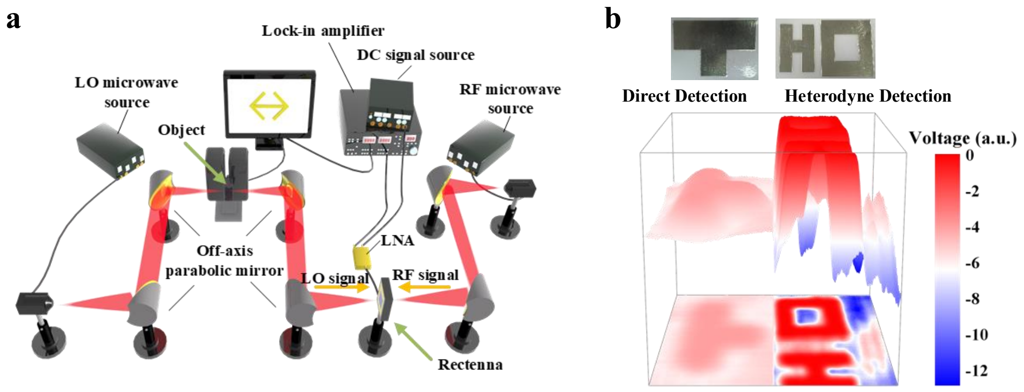

Supplementary Figure S7. Schematic diagram of the terahertz heterodyne imaging setup (a) and results (b). Contrast image obtained from direct detection (Left) and heterodyne detection for scanned THz imaging (Right). By performing 3D visualization processing on the collected voltage data and normalizing, it is evident that heterodyne imaging exhibits higher efficiency.

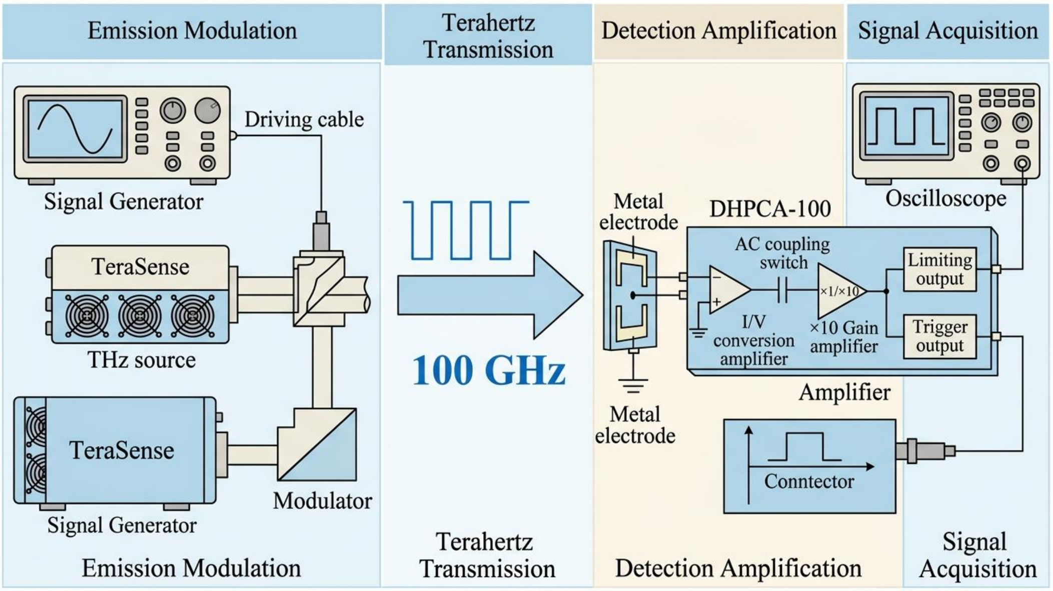

Supplementary Figure S8. Schematic diagram of the terahertz high-speed modulation and detection system.

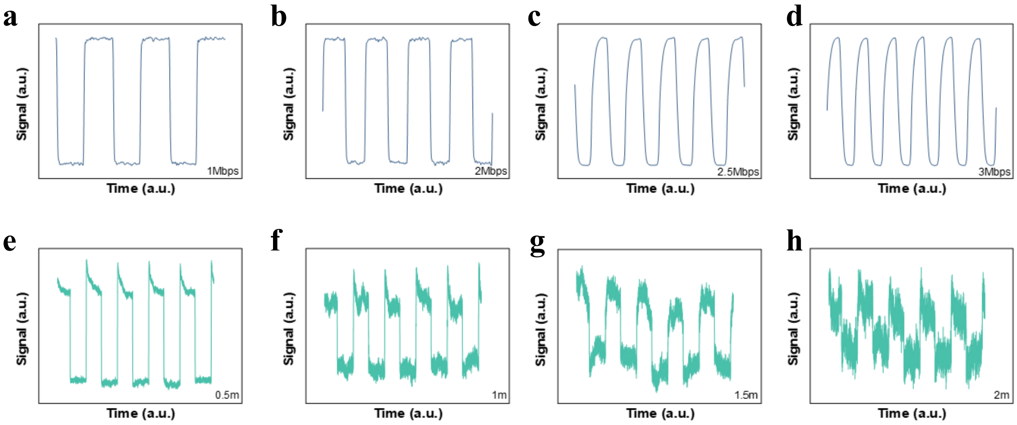

Supplementary Figure S9. Heterodyne communication performance versus transmission rate (a-d) and distance (e-h).
